# Cyclodextrin Inclusion Complexes of Auranofin and Its Iodido Analog: A Chemical and Biological Study

**DOI:** 10.3390/pharmaceutics13050727

**Published:** 2021-05-15

**Authors:** Damiano Cirri, Ida Landini, Lara Massai, Enrico Mini, Francesca Maestrelli, Luigi Messori

**Affiliations:** 1 Department of Chemistry and Industrial Chemistry, University of Pisa, Via Moruzzi 13, 56124 Pisa, Italy; damiano.cirri@dcci.unipi.it; 2 Department of Health Sciences, University of Florence, Viale Pieraccini 6, 50139 Firenze, Italy; ida.landini@unifi.it; 3 Department of Chemistry “Ugo Schiff”, University of Florence, Via della Lastruccia 3-13, 50019 Sesto Fiorentino, Italy; lara.massai@unifi.it

**Keywords:** auranofin, metal-based drugs, cyclodextrin encapsulation

## Abstract

Auranofin (AF) and its iodido analog, i.e., Au(PEt_3_) I (AFI), were reported to exhibit very promising anticancer properties both in vitro and in vivo. However, both these gold compounds have a scarce aqueous solubility that hampers their pharmaceutical use. Here, we explore whether encapsulation of these metallodrugs inside hydroxypropyl-beta–cyclodextrin (HPβ–CD) may lead to an improved biopharmaceutical profile for the resulting adducts. Phase solubility studies, performed at 25 °C in an aqueous buffer, revealed, in both cases, the formation of a 1:1 drug to cyclodextrin complex; a far greater apparent stability constant (K_1:1_) was measured for AFI compared to AF (331 M^−1^ versus ca. 30 M^−1^). NMR studies conducted on the AFI/HPβ–CD system confirmed the formation of a stable 1:1 adduct. Then, binary systems of AF and AFI with HPβ–CD were prepared by colyophilization and characterized by DSC and PXRD. The results revealed the occurrence of drug complexation and/or amorphization for the AFI/HPβ–CD binary system. Afterwards, the antiproliferative properties of the two cyclodextrin adducts and of the corresponding free drugs were comparatively evaluated in vitro in three representative ovarian cancer cell lines, i.e., A2780, SKOV3, and IGROV-1. The results, in all cases, point out that CD complexation of the two gold drugs does not substantially affect their biological activity. The implications of these findings are discussed in the frame of the current knowledge of AF and its analogs.

## 1. Introduction

**Figure 1:**
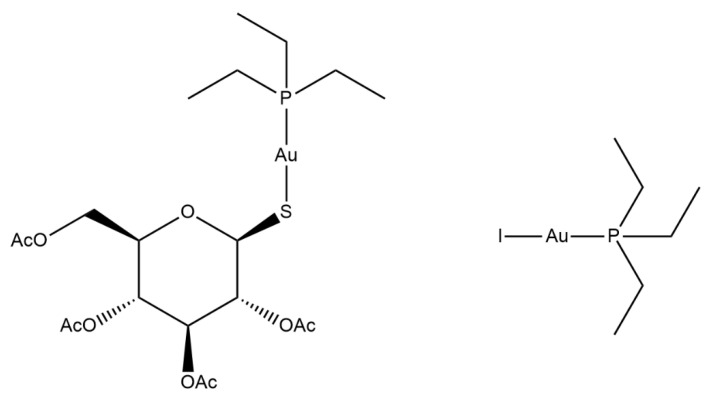
From left, auranofin and its iodide derivative, Au (PEt_3_) I.

Since the discovery of the anticancer activity of cisplatin in the early sixties and its subsequent approval by the FDA in 1978, metal-based drugs have gained growing attention and a role in the field of medicinal chemistry. For this reason, many other metal-based compounds have been prepared and screened in the last few decades as therapeutic agents against a variety of ailments, with some relevant results. Among the investigated metal-based drugs, auranofin (AF hereafter; Figure 1, left), a gold (I) compound for oral administration, is undoubtedly one of the most promising molecules. Indeed, AF is an already established drug that has been in clinical use since 1985 for the treatment of some severe forms of rheumatoid arthritis. In recent years, owing to the increased interest toward drug repurposing approaches, AF has reached the clinical trial stage for the treatment of various malignancies as well as a few parasitic diseases [1]. 

In the frame of the structure-activity studies of gold(I) drugs carried out in our laboratory, a number of halido and pseudohalido derivatives of auranofin have been designed and synthesized and subsequently tested as antibacterial and anticancer drug candidates [2,3]. Among these, the iodido derivative of auranofin, i.e., Au (PEt_3_)I (AFI hereafter; Figure 1, right), showed a significantly improved biodistribution profile in vivo and excellent activity against an orthotopic model of ovarian cancer [4]. 

However, despite their attractive features, many metallodrugs may manifest scarce aqueous solubility, and this can obviously hinder pharmaceutical use. This is the case of the gold compounds investigated here, with AF reported to be hydrophobic and lipid-soluble [5] and AFI exhibiting an even worse solubility profile due to the presence of the iodine atom and greater lipophilicity. Accordingly, a smart strategy to improve both the aqueous solubility [6,7] and pharmacological performance (i.e., in vivo biodistribution and cellular uptake) of AF and its analogs might consist of its encapsulation inside cyclodextrins.

Cyclodextrins are cyclic (α-1,4)-linked oligosaccharides of d-glucopyranose, originating from the enzymatic degradation of starch. Cyclodextrins are “cage molecules”: the core of their structure is composed of a dimensionally stable hydrophobic cavity that is able to trap or encapsulate other molecules. The remarkable encapsulation properties of cyclodextrins give rise to a peculiar “host–guest” chemistry that has the potential to greatly modify the physical, chemical, and/or biological characteristics of the guest molecule. In the field of pharmaceutics, of particular interest are the inclusion complexes formed by cyclodextrins with poorly water-soluble drugs that may improve their stability, solubility, dissolution rate, and bioavailability [8]. 

Accordingly, we report here on the encapsulation of AF and AFI inside (2-hydroxypropyl)-β–cyclodextrin (HPβ–CD). The choice of the latter was dictated by the encapsulation capacity of such a β-cyclodextrin derivative towards lipophilic drugs and by its safety; indeed, this cyclodextrin is currently a component of several marketed formulations [9]. HPβ–CD is recognized as an inactive pharmaceutical ingredient that is accepted for parenteral use by the FDA [10]. The resulting adducts, prepared by freeze-drying, were first characterized and then tested in vitro to assess their antiproliferative properties toward three different ovarian cancer cell lines. 

## 2. Materials and Methods

### 2.1. Chemicals

AF was purchased from Euroclone S.p.a. (Milan, Italy). Hydroxypropyl-β–cyclodextrin (HPβ–CD, average molecular weight 1399, MS 0.65) was a generous gift of Roquette Pharma (Alessandria, Italy). AFI was synthesized according to the procedure reported in [3]. 

### 2.2. Analytical Methods for Drug Determination

AF was analytically determined in aqueous solution by HPLC-UV at 240 nm using a Merck Hitachi Elite La Chrom with a UV–vis detector. AFI was dissolved in EtOH/water 1:5 *v*/*v*, and 5 different dilutions (from 0.2831 to 0.02831 mg/mL) were prepared in order to obtain the standard. For each sample, 20 µL were injected using a Zorbax CN (4.6 × 150 mm, 5 µm, Agilent, Santa Clara, CA, USA) maintained at 40 °C and the mobile phase was a mixture of methanol and NaH_2_PO_4_ 0.01 M at a 60:40 *v/v* ratio with a flux of 0.6 mL/min. The linearity, calculated with three injections for each level, was above 0.999. LOQ = 0.01586 mg/mL; LOD = 0.0064 mg/mL.

AFI was spectrophotometrically evaluated with a 1601 UV–vis spectrophotometer (Shimadzu Italia s.r.l., Milano, Italy) at 237.8 nm. The standard curve was prepared by dissolving AFI in EtOH/water 1:5 *v*/*v*. The linearity was determined on five concentration levels (from 0.12175 to 0.02435 mg/mL), with three injections for each level. The coefficient of linear correlation was above 0.999. LOQ = 0.01346 mg/mL; LOD = 0.0044 mg/mL.

### 2.3. Phase Solubility Studies

For phase solubility, an excess of drugs was added to 10 mL of water containing increasing amounts of HPβ–CD (0–25 mM). The samples were sealed, sonicated for 60 min (Eurosonic 44, Wilten Woltil, de Meern, The Netherlands), and then magnetically stirred at 25 ± 0.5 °C for 72 h. When complexation equilibrium was reached, the samples were withdrawn, filtered through 0.45 μm Millipore membrane filters, and spectrophotometrically assayed for drug content. Each experiment was performed in triplicate (coefficient of variation, CV < 5%). The apparent stability constants (K_1:1_) of the drug/HPβ–CD complexes were calculated from the slope of the straight line of phase solubility diagrams and drug solubility in the absence of HPβ–CD (S_0_) [11].

### 2.4. NMR Studies

NMR studies were performed on a Bruker equipment (Billerica, MA, USA). More precisely, the analysis were conducted with an Avance III 400 spectrometer equipped with a Ultrashield 400 Plus magnet (resonating frequency 400.13 for ^1^H) and a 5 mm PABBO BB-1H/D Z-GRD probe. ^31^PNMR characterization spectrum was performed in CDCl_3_ with a standard 90-degrees power-gated pulse sequence (zgpg). ROESY spectrum was recorded through a phase-sensitive pulse sequence, with a continuous wave spinlock for mixing and water suppression using excitation sculpting with gradients (roesyesgpph). The sample was prepared as a solution of AFI (0.4 mM) and HPβ–CD (5 mM) in degassed D_2_O. The experiment was performed with internal stabilization of temperature to 28 °C to avoid artifact generation. The acquisition parameters were: 16 scans; 512 increments; spinlock time 400 ms; recycle delay 2.5 s; transmitter frequency offset 4.79 ppm. All deuterated solvents were purchased from Sigma-Aldrich (Darmstadt, Germany) with a deuteration degree of 99.8%. All the spectra were calibrated on the solvent residual peak. 

### 2.5. Binary Systems Preparation Methods

The 1:1 mol/mol drug/HPβ–CD physical mixtures (P.M. hereafter) were prepared by the homogeneous mixing of previously weighed powders in a mortar with a spatula for 15 min. For the preparation of colyophilized products (COL), aqueous solutions of equimolar drug/HPβ–CD were prechilled and then freeze-dried (−50 °C, 1.3 × 10^−2^ mmHg) with a Leybold-Heraeus (Cologne, Germany) Lyovac GT2. The freeze-dried product was sieved, and a 75–150 μm granulometric sieve fraction was used for the following tests. 

### 2.6. Differential Scanning Calorimetry (DSC)

Thermographs of the individual components or drug/HPβ–CD products were obtained with a Mettler TA4000 calorimeter equipped with a DSC25 cell (Columbus, OH, USA). The samples, weighed with a Mettler M3 Microbalance (5–10 mg), were scanned in Al pans pierced with a perforated lid at 10 °C/min, from 30 to 150 °C, under static air.

### 2.7. Powder X-Ray Diffractometry (PXRD)

A Bruker D8-advance (Billerica, MA, USA) X-ray diffractometer was employed for the diffraction patterns of drugs and drug–cyclodextrin binary systems. The parameters were: Cu Kα radiation, voltage 40 kV, current 55 mA, and 2θ over a 5–40° range at a scan rate of 0.05°/s. A Sol-X^®^ solid-state Si (Li) was used as a detector, and C/Ni Goebel-Spiegel mirrors in the incident beam were used as a monochromator; 1.0 mm divergence, 0.2 scatter, and 0.1 for the receiving slits were used. All samples were examined at room temperature.

### 2.8. Cell Lines and Culture Conditions

The human ovarian cancer cell lines used in the present study (i.e., A2780, IGROV1, and SKOV3) were obtained from American Type Culture Collection (ATCC). All cell lines were maintained in RPMI-1640 medium supplemented with 10% of fetal calf serum at 37 °C in a 5% CO_2_ humidified atmosphere and subcultured twice weekly. The culture medium was supplemented with antibiotics (i.e., penicillin 100 U/mL and streptomycin 100 μg/mL).

### 2.9. Cytotoxicity Assays

Cell proliferation inhibitions induced by AF, AFI and its counterpart conjugated to HPβ–CD (AF/HPβ–CD and AFI/HPβ–CD), and HPβ–CD alone were evaluated against the abovementioned ovarian cancer cell lines, according to the method described by Skehan et al. [12]. Exponentially growing cells were seeded in 96-well microplates at a density of 8 × 10^3^ for 24 h prior to the addition of the study compounds. After 24 h, the medium was removed and replaced with fresh medium containing concentrations of the investigated compounds, ranging from 0.03 to 100 µM, and incubated for 72 h. After 72 hours of drug exposure, the cells were fixed with trichloroacetic acid and stained with a sulforhodamine B/acetic acid solution. The sulforhodamine B fixed to the cells was dissolved in Tris-HCl, and absorbance was read on an automated plate reader at a wavelength of 540 nm. IC_50_ drug concentration was determined as a 50% reduction in the net protein content in the drug-treated cells (as measured by sulforhodamine B staining) compared to untreated control cells. All the reported IC_50_ values represent the mean of three independent experiments. 

## 3. Results and Discussion

### 3.1. Phase Solubility Studies

**Figure 2:**
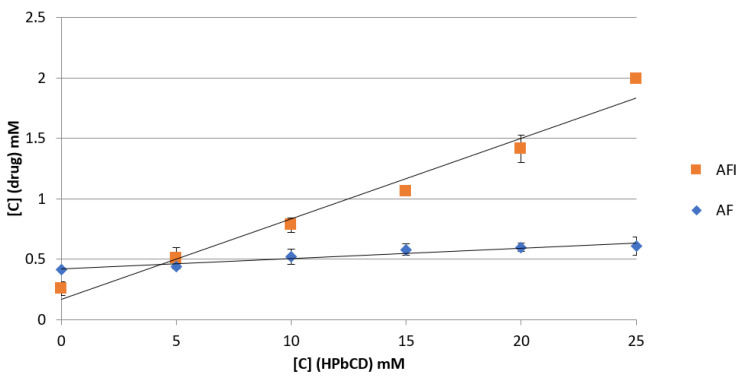
Phase solubility studies of AFI and AF with HPβ–CD at 25 °C in water.

Phase solubility measurements conducted on the two gold drugs (see Figure 2) revealed, in both cases, that the solubility of the drug linearly increases as a function of cyclodextrin concentration, showing an AL-type diagram according to the classification of Higuchi and Connors [11]. This behavior is indicative of the formation of a 1:1 molar complex in solution [11]. In the case of AF, the calculated equation was y = 0.012x + 0.418 (R^2^ = 0.975), so an apparent stability constant of about 30 M^−1^ was determined for the AF/HPβ–CD adduct. 

A far greater value of the apparent stability constant (K_1:1_~331 M^−1^) was determined for AFI (y = 0.068x + 0.169; R^2^ = 0.973), highlighting a stronger interaction between the latter drug and the hydrophobic cavity of HPβ–CD. This behavior is related to the different affinity of such drugs for HPβ–CD, arising from their different lipophilicities; indeed, the LogP of AFI is reported to be 4.6, to be compared with a value of 1.6 reported for auranofin [2]. Accordingly, AFI shows lower water solubility (0.22 ± 0.05 mM) than AF (0.41 ± 0.01 mM) but a greater affinity for the cyclodextrin hydrophobic cavity that leads to a 10-fold increase of water solubility in the presence of 25 mM of HPβ–CD.

### 3.2. NMR Studies on the AFI/HPβ–CD System

**Figure 3:**
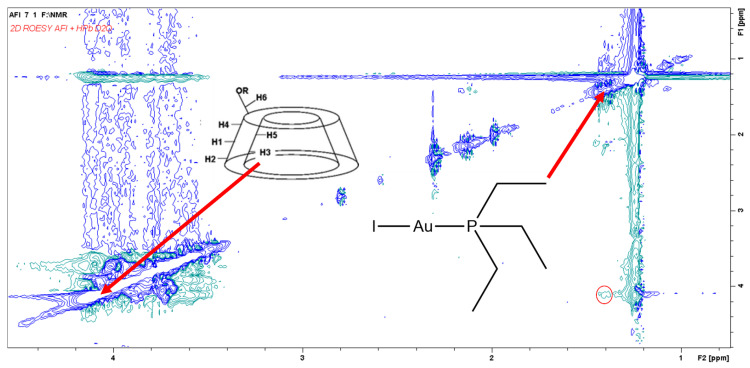
ROESY experiment performed on the abovementioned AFI/HPβ–CD mixture. The circled cross peak is indicative of the formation of an inclusion supramolecular adduct.

We decided to confirm the internalization of AFI inside the cavity of this cyclodextrin through the detection of an “intermolecular” nuclear Overhauser effect between AFI and HPβ–CD, which is strictly related to the spatial distance between the involved atoms. This determination was performed through a nonselective two-dimensional ROESY experiment, in which the formation of an AFI/HPβ–CD internalization adduct was confirmed through detection of the cross peak highlighted in Figure 3, the latter being related to a close-in-space dipolar interaction between the methyl groups of AFI (1.34 ppm) and the H3 protons of HPβ–CD (4.04 ppm) [13].

### 3.3. Binary System Characterization in the Solid State

**Figure 4:**
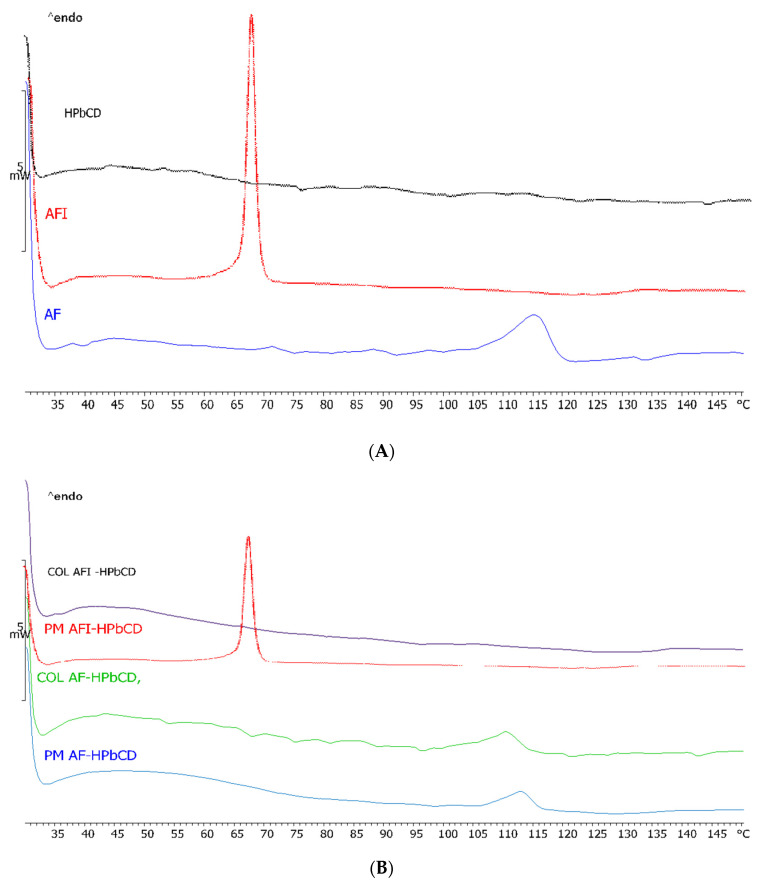
DSC thermographs of the raw materials (**A**) and the respective 1:1 M binary systems (**B**).

To obtain further insight into possible solid-state interactions between the two components, equimolar drug/HPβ–CD binary systems were prepared by colyophilization and characterized by DSC and PXRD analysis in comparison with the simple physical mixtures and the raw materials. The thermographs of the raw materials are reported in Figure 4A.

**Figure 5:**
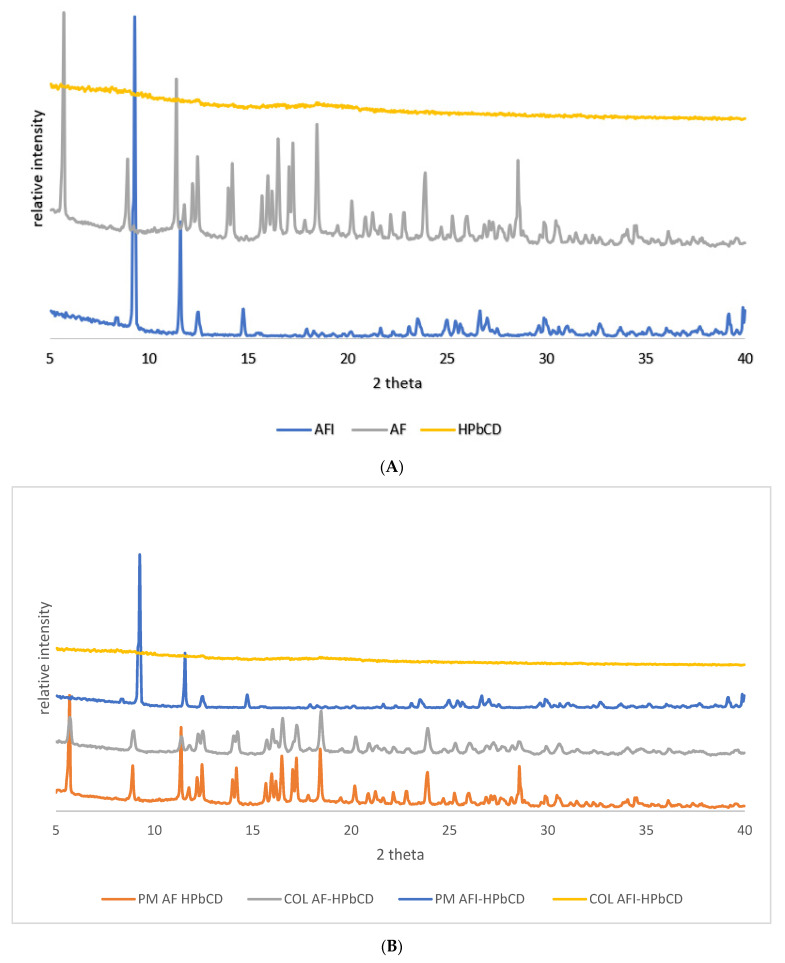
PXRD graphs for the raw materials (**A**) and the relative 1:1 M binary systems (**B**).

As shown in Figure 4, the thermal curve of pure HPβ–CD did not exhibit any significant phenomena in the studied range; at variance—in line with expectations—AF showed an endothermic peak attributable to drug melting at 115.17 °C and a melting enthalpy of 30.78 Jg^−1^ according to Kerç et al. [14]. The thermographs of AFI, which have never been described before, show an anhydrous profile and a melting peak at 67.26 °C with a melting enthalpy of 45.07 Jg^−1^, indicating its crystalline nature. In Figure 4B, the thermal profiles of the equimolar binary systems obtained by freeze-drying and the relative physical mixtures are reported. As shown, for both AF/HPβ–CD P.M. and COL, just a slight reduction of the melting temperature (112.80 and 110.27 °C) and enthalpy (12.2 and 10.8 Jg^-1^) was observed, indicative of scarce interaction between AF and HPβ–CD. The same was observed for P.M. AFI/HPβ–CD (65.43 °C; 45.07 Jg^−1^), while the COL AFI/HPβ–CD thermal profile showed complete disappearance of the drug melting peak. This behavior is indicative of complete amorphization and/or complexation in the HPβ–CD matrix as a consequence of the freeze-drying process [15]. The X-ray powder diffraction patterns are reported in Figure 5. 

In Figure 5A, the PXRD patterns of the raw materials are reported. HPβ–CD shows the typical amorphous profile, while the drugs present typical diffraction peaks. The diffraction peaks, attributable to drug crystals, were well detected for both AF and AFI physical mixtures and COL with AF, as reported in Figure 5B. In contrast, a completely diffused pattern was obtained for COL with AFI, thus confirming the complete amorphization and/or complexation of AFI in the HPβ–CD matrix as a result of stronger interaction between the components, depending on the preparation method. These results agree with the DSC analysis, thus confirming the occurrence of drug amorphization.

### 3.4. Biological Properties

The cytotoxic effects of AF and AFI, as well as their conjugates with cyclodextrins, (AF/HPβ–CD and AFI/HPβ–CD) and of HPβ–CD alone, taken as a control, were subsequently evaluated in three different ovarian cancer cell lines, i.e., A2780, SKOV3, and IGROV-1. After 72 h of exposure, no cytotoxic activity was observed for HPβ–CD (IC_50_ > 100 µM; data not shown). In line with expectations, auranofin and its iodide derivative showed remarkable and roughly similar cell growth inhibitory properties in the three tested human ovarian cancer cell lines (IC50 < 1 µM). These values are in accord with those reported in a previous study by our group [16]. No substantial differences in the cytotoxic effects of AFI/HPβ–CD compared to unconjugated AFI were observed in the three cell lines; on the other hand, AF/HPβ–CD, in comparison to the free drug, was found to exert somewhat lower cytotoxic effects against A2780 and IGROV-1 cells but similar antiproliferative effects against SKOV3 cells (Table 1).

## 4. Conclusions

In recent years, a variety of gold(I) compounds, including AF and AFI, have been found to manifest promising anticancer properties both in vitro and in vivo toward several cancer types, thus attracting the attention of medicinal chemists. Remarkably, auranofin, owing to its favorable pharmacological profile and the opportunities offered by drug repurposing, has recently been admitted to cancer clinical trials. As both gold compounds are characterized by a rather scarce solubility profile in water, we thought that suitable pharmaceutical strategies might be implemented to address this point and increase their solubility in aqueous solutions. One of the approaches typically exploited to overcome these solubility issues is to design and develop adequate drug delivery systems for the transport of these compounds. By functionalization, encapsulation, or formulation of the metal complexes, several types of drug delivery systems have been reported to improve the pharmacological profile of metal-based drugs, increasing their overall stability, bioavailability, and anticancer activity and reducing their toxicity towards normal cells. Here, we have explored whether encapsulation within cyclodextrins might offer an appropriate strategy to achieve a better biopharmaceutical and pharmacological profile for these gold drugs. Indeed, cyclodextrins are “cage molecules” with a large hydrophobic cavity that is able to encapsulate other molecules. In our case, hydroxypropyl-beta-cyclodextrin (HPβ–CD) turned out to be a suitable system for the encapsulation of auranofin (AF) and its iodido analog (AFI). Phase solubility studies, performed at 25 °C in an aqueous buffer, revealed, in both cases, the formation of a stable 1:1 drug to cyclodextrin complex; a far greater apparent stability constant (K_1:1_) was measured for AFI compared to AF (331 versus ca. 30 M^−1^), in agreement with the larger lipophilicity of AFI. In turn, NMR studies conducted on the AFI/HPβ–CD system confirmed the formation of the 1:1 adduct through the detection of a characteristic ROESY cross peak between the protons belonging to the two components of the binary system. Then, adducts of AF and AFI with HPβ–CD were further prepared and characterized by DSC and PXRD. The results confirmed the occurrence of drug complexation and/or amorphization for the AFI/HPβ–CD binary system. Anyway, owing to the relatively low stability constants of the adducts that we measured, the gold drug/cyclodextrin complexes are expected to dissociate to a large extent in biofluids and to be in equilibrium with a substantial amount of the free drug. Indeed, the antiproliferative properties of the two cyclodextrin adducts turned out to roughly reproduce the cytotoxic properties of the free gold complexes. Despite the fact that no enhancement of antiproliferative actions was disclosed for AFI or AF, these results suggest that inclusion in an appropriate cyclodextrin may be a suitable strategy to improve the aqueous solubility of these hydrophobic gold compounds. The great increase in solubility, highlighted for AFI in comparison to AF upon complexation, suggests a relevant contribution of the LogP value to the efficiency of the encapsulation process. The increased solubility of AFI, obtained with HPβ–CD without the use of any solvent, opens the possibility of using this molecule in clinical trials and in therapy with a wider dosage range. In conclusion, the utilization of inclusion complexes with approved cyclodextrins could represent a cheap and suitable way for administering hydrophobic auranofin-like complexes without affecting their pharmacological activity. Yet, a final comment is due concerning the binding of these specific gold compounds to serum proteins. Many earlier publications suggested that these gold compounds readily interact with serum albumin [17,18]: after parenteral administration, these drugs are kept in solution through serum albumin interaction, regardless of the presence of cyclodextrins. Moreover, it was previously reported that these parenterally administered gold compounds retain their biological activity despite their extensive binding to serum proteins [4], confirming the general importance and intertest of these novel drug candidates.

## Figures and Tables

**Table 1:** Inhibitory effects of auranofin, AFI, AF/HPβ–CD, and AFI/HPβ–CD on cell growth of the studied tumor cell lines after 72 h of compound exposure. Data obtained as a mean of three independent experiments.

	IC_50_ Mean ± SD (μM)			
	Auranofin	Auranofin/HPβ–CD	AFI	AFI/HPβ–CD
A2780	0.611 ± 0.085	0.831 ± 0.079	0.898 ± 0.117	0.910 ± 0.162
SKOV3	0.360 ± 0.004	0.577 ± 0.177	0.685 ± 0.100	0.535 ± 0.188
IGROV-1	0.657 ± 0.164	1.092 ± 0.045	0.856 ± 0.018	0.684 ± 0.158

## Data Availability

The data presented in this work are available under request to the corresponding authors.
